# Role of adjuvant chemoradiotherapy and chemotherapy in patients with resected gallbladder carcinoma: a multi-institutional analysis (KROG 19-04)

**DOI:** 10.20892/j.issn.2095-3941.2020.0667

**Published:** 2021-12-20

**Authors:** Sung Uk Lee, Jinsil Seong, Tae Hyun Kim, Jung Ho Im, Woo Chul Kim, Kyubo Kim, Hae Jin Park, Tae Gyu Kim, Youngkyong Kim, Bae Kwon Jeong, Jin Hee Kim, Byoung Hyuck Kim, Taek-Keun Nam

**Affiliations:** 1 Center for Proton Therapy and Center for Liver and Pancreatobiliary Cancer, National Cancer Center, Goyang 10408, Korea; 2 Department of Radiation Oncology, Yonsei Cancer Center, Yonsei University College of Medicine, Seoul 03722, Korea; 3 Department of Radiation Oncology, CHA Bundang Medical Center, CHA University School of Medicine, Seongnam 13497, Korea; 4 Department of Radiation Oncology, Inha University Hospital, Inha University School of Medicine, Incheon 22332, Korea; 5 Department of Radiation Oncology, Ewha Womans University Mokdong Hospital, Ewha Womans University College of Medicine, Seoul 07985, Korea; 6 Department of Radiation Oncology, Hanyang University Medical Center, Hanyang University College of Medicine, Seoul 04763, Korea; 7 Department of Radiation Oncology, Samsung Changwon Hospital, Sungkyunkwan University School of Medicine, Changwon 51353, Korea; 8 Department of Radiation Oncology, Kyung Hee University Hospital, Kyung Hee University College of Medicine, Seoul 02447, Korea; 9 Department of Radiation Oncology, Gyeongsang National University Hospital, Gyeongsang National University College of Medicine, Jinju 52727, Korea; 10 Department of Radiation Oncology, Dongsan Medical Center, Keimyung University School of Medicine, Daegu 41931, Korea; 11 Department of Radiation Oncology, Seoul Metropolitan Government Seoul National University Boramae Medical Center, Seoul 07061, Korea; 12 Department of Radiation Oncology, Chonnam National University Hwasun Hospital, Chonnam National University College of Medicine, Hwasun 58128, Korea

**Keywords:** Gallbladder cancer, adjuvant treatment, chemoradiotherapy, locoregional recurrence-free survival, overall survival

## Abstract

**Objective:**

The effectiveness of adjuvant treatments for resected gallbladder carcinoma (GBC) has remained unclear due to lack of randomized controlled trials; thus, the aim of present study was to evaluate the role of adjuvant treatments, including chemoradiotherapy (CRT) and/or chemotherapy (CTx), in patients with resected GBC.

**Methods:**

A total of 733 GBC patients who received curative-intent surgical resection were identified in a multi-institutional database. Of 733 patients, 372 (50.8%) did not receive adjuvant treatment, whereas 215 (29.3%) and 146 (19.9%) received adjuvant CTx and CRT, respectively. The locoregional recurrence-free survival (LRFS), recurrence-free survival (RFS), and overall survival (OS) of the adjuvant treatment groups were compared according to tumor stage (stage II *vs.* stage III–IV).

**Results:**

In stage II disease (*n* = 381), the 5-year LRFS, RFS, and OS were not significantly different among the no-adjuvant therapy, CTx, and CRT groups, and positive resection margin, presence of perineural invasion, and Nx classification were consistently associated with worse LRFS, RFS, and OS in the multivariate analysis (*P* < 0.05). For stage III–IV (*n* = 352), the CRT group had significantly higher 5-year LRFS, RFS, and OS than the no-adjuvant therapy and CTx groups (67.8%, 45.2%, and 56.9%; 37.9%, 28.8%, and 35.4%; and 45.0%, 30.0%, and 45.7%, respectively) (*P* < 0.05).

**Conclusions:**

CRT has value as adjuvant treatment for resected GBC with stage III–IV disease. Further study is needed for stage II disease with high-risk features.

## Introduction

Gallbladder carcinoma (GBC) is an aggressive tumor originating from the biliary tract and is known to have an unfavorable prognosis^1^. Surgical resection is known as the only curative treatment for localized GBC, but a relatively high recurrence rate after surgery remains a major concern^2–5^. Theoretically, adjuvant chemoradiotherapy (CRT) or chemotherapy (CTx) would be helpful to control the subclinical locoregional and systemic tumor burden after surgical resection; thus, the addition of adjuvant treatments might be a reasonable approach for resected GBC. However, although GBC is a malignancy of a subsite of the biliary tract and its natural course or etiology is distinctive from that of other subsites, most previous studies assessing the efficacy of adjuvant treatments in resected GBC included relatively small and heterogeneous populations, including GBC, intrahepatic and extrahepatic bile duct cancer and/or periampullary cancer^2,6–13^. The SWOG S0809 trial showed that adjuvant capecitabine and gemcitabine followed by CRT in resected GBC was feasible and effective, but it did not compare this treatment strategy with surgery alone^14^. Thus, the effectiveness of adjuvant treatments, including CRT or CTx, for resected GBC has remained unclear due to a lack of randomized controlled trials. Unfortunately, due to the rarity of GBC, conducting randomized controlled trials to evaluate the effectiveness of adjuvant treatments for resected GBC patients in the real world is difficult. For these reasons, the present study was designed to evaluate the role of adjuvant treatments, including CRT and/or CTx, in resected GBC compared with surgical resection alone using the multi-institutional database of the Korean Radiation Oncology Group (KROG) and to identify subgroups that are likely to benefit from adjuvant treatments by analyzing the clinicopathologic factors associated with recurrence and survival.

## Materials and methods

### Patients

The data of GBC patients who underwent primary surgical resection with curative intent at any of the 12 KROG member institutions between October 2001 and October 2017 were reviewed. The inclusion criteria of the present study were as follows: histologically confirmed adenocarcinoma of the gallbladder; pathologic T2 or higher disease, assessed by the American Joint Committee on Cancer (AJCC) staging system (8th edition); patients who underwent curative-intent surgical resection, defined as the eradication of the whole tumor(s) without gross residual disease; no distant metastasis; and no history of neoadjuvant treatment before surgery or previous or current malignancy. The clinicopathologic data of each patient, including age, gender, histologic findings, stage, serum carbohydrate antigen 19-9 (CA 19-9), surgical procedures, adjuvant treatments, sites and time of recurrence and survival, were collected. The collected data were managed by assigning case numbers to each participating institute and anonymizing them. Data analysis was performed centrally at the National Cancer Center, Republic of Korea, and all methods were performed in accordance with the relevant guidelines and regulations. This study was approved by the institutional review board of each participating institute and KROG (Approval No. KROG 19-04) and complied with the Declaration of Helsinki and Good Clinical Practice guidelines. The requirement for written informed consent was waived due to the retrospective nature of the study.

## Assessments and statistical analysis

Disease recurrence was confirmed pathologically and/or radiologically with evidence of an increase in size over time. Locoregional recurrence was defined as newly appearing or reappearing tumor(s) within the tumor bed and regional lymphatic area, including the porta hepatis, peripancreatic region, celiac region, origin of the superior mesenteric artery and para-aortic nodes, and distant recurrence was defined as newly appearing tumor(s) in distant organs or nonregional lymph nodes. Locoregional recurrence-free survival (LRFS) and recurrence-free survival (RFS), defined as the time from the date of surgical resection to the date of locoregional recurrence and any type of recurrence, respectively, and these were censored at the date of the last follow-up if the patients had no evidence of recurrence. Overall survival (OS) was defined as the time from the date of surgical resection to the date of death from any cause. Survival outcomes were estimated using the Kaplan–Meier method and compared using the log-rank test. In multivariate analysis, hazard ratios (HRs) were estimated using a Cox proportional hazards model. Statistical analyses were performed with STATA version 14.0 (StataCorp, College Station, TX, USA); all tests were 2-sided, and a *P*-value < 0.05 was considered statistically significant.

## Results

A total of 745 patients who met the inclusion criteria were identified from the multi-institutional database. Of them, 12 patients who received adjuvant radiotherapy without concurrent CTx were excluded, and the remaining 733 patients were analyzed in the present study. Tumor stage was stage II in 381 (52.0%) patients, stage III in 299 (40.8%) patients, and stage IV in 53 (7.2%) patients. Regarding the operative procedures, of 733 patients, 621 (84.7%) and 112 (15.3%) patients underwent radical cholecystectomy and cholecystectomy, respectively; 656 (89.5%) patients underwent lymph node dissection, with a median number of dissected lymph nodes of 9 (range, 1–60), and 77 (10.5%) patients did not undergo lymph node dissection. After surgical resection, 372 (50.8%) patients did not receive adjuvant treatment (No-AT), and 215 (29.3%) and 146 (19.9%) patients received adjuvant CTx and CRT, respectively. In the CTx group, 5-fluorouracil (5-FU)-based regimens (*n* = 143, 66.5%), gemcitabine-based regimens (*n* = 68, 31.6%), and combined 5-FU and gemcitabine (*n* = 4, 1.8%) regimens were administered, with a median number of cycles of 6 (range, 1–24). In the CRT group, a median radiation dose of 50.4 Gy (range, 45.0–54.0 Gy), with a daily fraction of 1.8–2.0 Gy, was delivered to the tumor bed and regional lymphatic area with concurrent 5-FU- (*n* = 136) or platinum/gemcitabine-based (*n* = 10) CTx.

The baseline characteristics of the 3 adjuvant treatment groups (No-AT, CTx, and CRT) are compared in **Table 1**. For stage II patients, the No-AT group had significantly more older patients than the CTx group and had significantly fewer patients with positive resection margins than the CRT group (*P* < 0.05) (**Table 1**). For stage III–IV patients, the No-AT group had significantly more older patients than the CTx and CRT groups and had significantly fewer patients with N1–N2 disease than the CTx and CRT groups (*P* < 0.05) (**Table 1**). The other baseline characteristics were not significantly different among the 3 groups (**Table 1**).

**Table 1:** Comparison of the baseline patient characteristics among the adjuvant treatment groups

Characteristics	Stage II (*n* = 381)				Stage III–IV (*n* = 352)			
	No-AT (*n* = 258)	CTx (*n* = 82)	CRT (*n* = 41)	*P* value	No-AT (*n* = 114)	CTx (*n* = 133)	CRT (*n* = 105)	*P* value
Gender, *n* (%)								
Male	113 (44)	33 (40)	22 (54)	0.364*	44 (39)	55 (41)	43 (41)	0.897*
Female	145 (56)	49 (60)	19 (46)		70 (61)	78 (59)	62 (59)	
Age (years)								
Median [range]	67 [29–87]	61 [29–88]	66 [44–81]	0.000^†^	71 [41–91]	62 [38–87]	63 [26–83]	0.000^†^
≤60 years, *n* (%)	53 (21)	36 (44)	10 (24)	0.000*	22 (19)	52 (39)	39 (37)	0.002*
>60 years, *n* (%)	205 (79)	46 (56)	31 (76)		92 (81)	81 (61)	66 (63)	
Tumor size (cm)								
Median [range]	2.3 [0.1–8.8]	2.7 [0.7–9.0]	2.5 [0.5–10.5]	0.420^†^	3.4 [1.0–13]	3.5 [0.9–13.0]	3.5 [1.0–9.0]	0.534^†^
≤3 cm, *n* (%)	79 (67)	38 (63)	22 (65)	0.846*	35 (43)	34 (44)	37 (47)	0.866*
>3 cm, *n* (%)	38 (33)	22 (37)	12 (35)		47 (57)	43 (56)	42 (53)	
T classification, *n* (%)								
T2	258 (100)	82 (100)	41 (100)	–	44 (39)	58 (44)	48 (46)	0.554*
T3–4	–	–	–		70 (61)	75 (56)	57 (54)	
N classification, *n* (%)								
Nx	52 (20)	11 (13)	3 (7)	0.075^‡^	8 (7)	2 (2)	1 (1)	0.027^‡^
N0	206 (80)	71 (87)	38 (93)		27 (24)	25 (19)	17 (16)	
N1					60 (53)	90 (68)	74 (71)	
N2					19 (17)	16 (12)	13 (12)	
Preop CA 19-9 level (U/mL)								
Median [range]	10 [0.1–2,400]	9 [0.8–383]	10 [2–119]	0.347^†^	21 [0.1–2,960]	21 [0.1–8,340]	19 [0.1–9,410]	0.370^†^
≤37 U/mL, *n* (%)	168 (83)	57 (84)	29 (85)	0.926*	59 (64)	81 (68)	60 (67)	
>37 U/mL, *n* (%)	35 (17)	11 (16)	5 (15)		34 (37)	39 (33)	29 (33)	
Postop CA 19-9 level (U/mL)								
Median [range]	8 [0.1–3,056]	8 [0.7–62]	9 [2.6–606]	0.639^†^	13 [0.1–3,487]	15 [0.1–850]	14 [0.1–1,445]	0.064^†^
≤37 U/mL, *n* (%)	204 (94)	72 (97)	34 (94)	0.543^‡^	70 (74)	104 (83)	78 (84)	0.131*
>37 U/mL, *n* (%)	13 (6)	2 (3)	2 (6)		25 (26)	21 (17)	15 (16)	
Histologic differentiation, *n* (%)								
WD/MD	224 (90)	69 (86)	36 (90)	0.639*	74 (70)	97 (76)	73 (72)	0.554*
PD	25 (10)	11 (14)	4 (10)		31 (30)	30 (24)	29 (28)	
Resection margin, *n* (%)								
Negative	249 (99)	72 (95)	38 (93)	0.025^‡^	97 (88)	118 (90)	96 (92)	0.599*
Positive	3 (1)	4 (5)	3 (7)		13 (12)	13 (10)	8 (8)	
LVSI, *n* (%)								
No	187 (79)	58 (82)	27 (67)	0.196*	38 (36)	51 (40)	51 (50)	0.122*
Yes	50 (21)	13 (18)	13 (33)		68 (64)	76 (60)	52 (50)	
PNI, *n* (%)								
No	193 (88)	43 (80)	31 (82)	0.199*	55 (54)	50 (43)	49 (49)	0.280*
Yes	26 (12)	11 (20)	7 (18)		47 (46)	66 (57)	52 (51)	
Extent of surgical resection, *n* (%)								
Non-radical	60 (23)	14 (17)	5 (12)	0.175*	20 (18)	7 (5)	6 (6)	0.001*
Radical	198 (77)	68 (83)	36 (88)		94 (82)	126 (95)	99 (94)	

No-AT, no adjuvant therapy; CTx, chemotherapy; CRT, chemoradiotherapy; Preop, preoperative; Postop, postoperative; CA 19-9, carbohydrate antigen 19-9; WD, well differentiation; MD, moderate differentiation; PD, poor differentiation; LVSI, lymphovascular space invasion; PNI, perineural invasion; Non-radical, cholecystectomy ± lymph node dissection; Radical, radical cholecystectomy and lymph node dissection. *Pearson’s chi-square test. ^†^One-way analysis of variance. ^‡^Fisher’s exact test.

**Figure 1:**
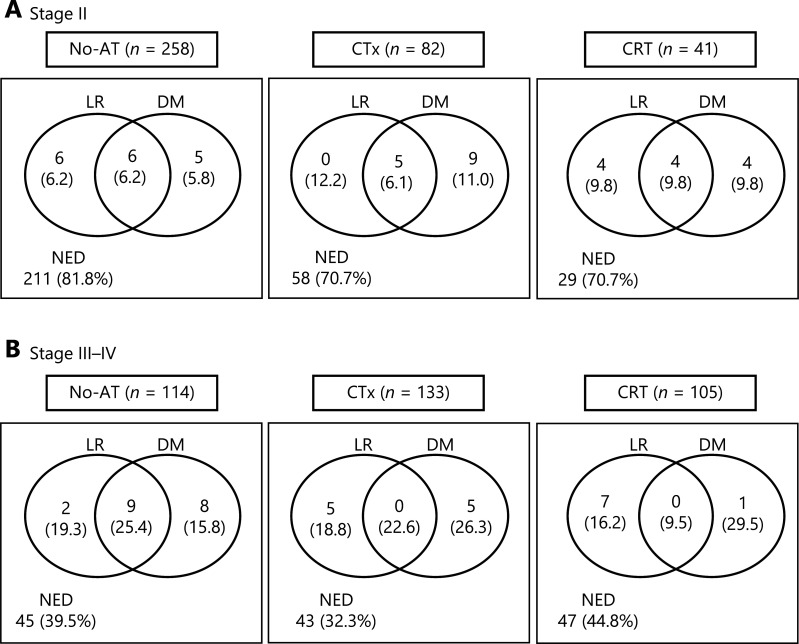
Patterns of failures among adjuvant treatment groups. (A) stage II, and (B) stage III–IV. LR, locoregional recurrence; DM, distant metastasis; NED, no evidence of disease; No-AT, no adjuvant treatment; CTx, chemotherapy; CRT, chemoradiotherapy.

The median follow-up durations of all patients and living patients were 39.8 months [interquartile range (IQR), 21.1–75.0 months] and 58.9 months (IQR, 30.4–91.5 months), respectively. At the time of analysis, a total of 268 deaths were observed, 224 (83.6%) of which were from disease progression. Disease recurrence was observed in 300 (40.9%) patients, and the patterns of failure were locoregional recurrence in 94 (12.8%) patients, distant metastasis in 112 (15.3%) patients, and both locoregional and distant recurrences in 94 (12.8%) patients. The proportions of patients who experienced disease recurrence in stages II and III–IV were 21.8% and 61.6%, respectively (**Figure 1**). The patterns of failure in stage II showed no differences among the 3 adjuvant treatment groups (*P* > 0.05) (**Figure 1A**). Regarding stage II disease, locoregional recurrence occurred in 12.4%, 18.3%, and 19.6% of the patients in the No-AT, CTx, and CRT groups, respectively, and distant metastasis occurred in 12.0%, 17.1%, and 19.6% (**Figure 1A**). In stage III–IV patients, the rate of locoregional recurrence was significantly lower in the CRT group (25.7%) than in the No-AT and CTx groups (44.7% and 41.4%, respectively, *P* = 0.002) (**Figure 1B**), and the rates of distant metastasis were similar among the 3 groups (41.2%, 48.9%, and 39.0% in the No-AT, CTx, and CRT groups, respectively, *P* = 0.233). In contrast, the CTx group showed no significant difference in the patterns of failures compared with the No-AT group (*P* = 0.234).

**Figure 2:**
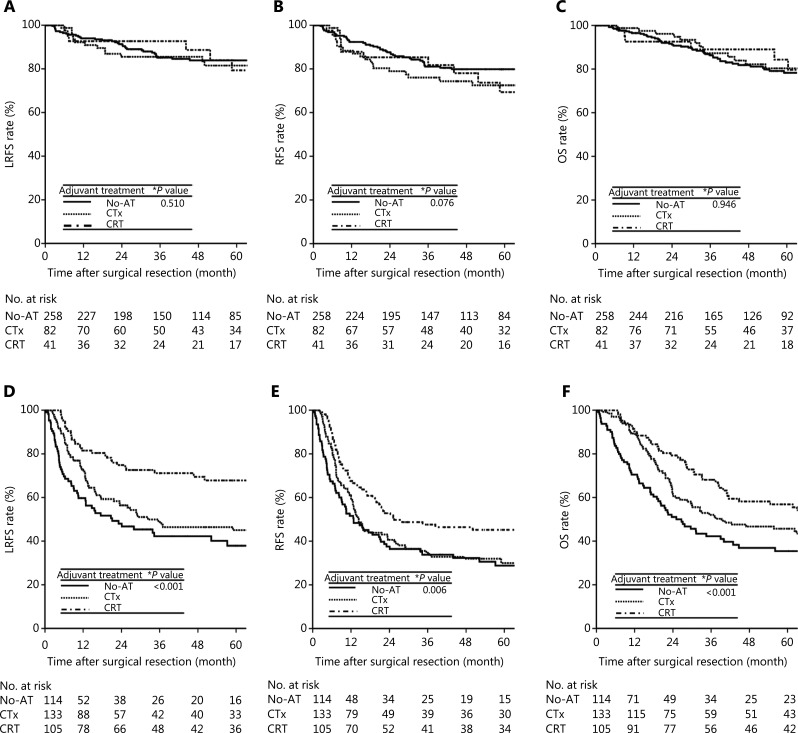
LRFS (A and D), RFS (B and E), and OS (C and F) curves among the adjuvant treatment groups in stage II and III–IV patients, respectively. LRFS, locoregional recurrence-free survival; RFS, recurrence-free survival; OS, overall survival; LR, locoregional recurrence; DM, distant metastasis; NED, no evidence of disease; No-AT, no adjuvant treatment; CTx, chemotherapy; CRT, chemoradiotherapy. *Log-rank test.

For all patients, the 5-year LRFS, RFS, and OS rates were 82.8%, 77.0%, and 79.3% in stage II disease and 50.2%, 34.4%, and 46.0% in stage III–IV disease, respectively. The univariate analyses of LRFS, RFS, and OS in stages II and III–IV were performed separately (**Table 2**). In patients with stage II disease, aged, Nx classification, preoperative CA 19-9 level > 37 U/mL, postoperative CA 19-9 level > 37 U/mL, presence of lymphovascular invasion, presence of perineural invasion, positive resection margin, and non-radical resection were associated with significantly worse LRFS, RFS, and OS (*P* < 0.05), and adjuvant treatments (CTx and CRT) were not significantly associated with LRFS, RFS, or OS compared with No-AT (**Figure 2A–C**) (**Table 2**). In patients with stage III–IV disease, advanced T and N classification, postoperative CA 19-9 level > 37 U/mL, poor differentiation, presence of lymphovascular invasion, and presence of perineural invasion were associated with significantly worse LRFS, RFS, and OS (*P* < 0.05), and the CRT group showed significantly better LRFS, RFS, and OS rates than the No-AT and CTx groups (*P* < 0.05) (**Figure 2D–F**) (**Table 2**). Younger patients showed significantly longer OS times than older patients, and patients with positive resection margins had significantly worse RFS and OS rates than patients with negative resection margins. Patients who received radical resection showed significantly longer LRFS and OS than patients who received non-radical resection. The results of multivariate analysis are summarized in **Table 3**. In patients with stage II disease, Nx classification, presence of perineural invasion, and positive resection margin were consistently significant factors associated with increased HRs for LRFS, RFS, and OS (*P* < 0.05). In patients with stage III–IV disease, advanced T and N classification and postoperative CA 19-9 level > 37 U/mL were significant factors associated with increased HRs for LRFS, RFS, and OS (*P* < 0.05). In addition, CRT significantly decreased the risks of LRFS [HR, 0.28; 95% confidence interval (CI), 1.25–0.51], RFS (HR, 0.52; 95% CI, 0.33–0.82), and OS (HR, 0.42; 95% CI, 0.25–0.70) compared with No-AT (*P* < 0.05), but CTx did not show statistical significance in decreasing the HRs for LRFS, RFS, and OS compared with No-AT (*P* > 0.05).

**Table 2:** Univariate analysis of LRFS, RFS, and OS according to tumor stage

Characteristics	*n*	Stage II (*n* = 381)						*n*	Stage III–IV (*n* = 352)					
		LRFS		RFS		OS			LRFS		RFS		OS	
		5-year (%)	*P* value*	5-year (%)	*P* value*	5-year (%)	*P* value*		5-year (%)	*P* value*	5-year (%)	*P* value*	5-year (%)	*P* value*
Gender														
Male	168	86.2	0.096	78.3	0.621	80.2	0.672	142	47.8	0.360	32.2	0.449	48.8	0.445
Female	213	80.1		76.0		78.4		210	52.0		36.1		44.2	
Age														
≤60 years	99	91.0	0.021	84.5	0.039	90.6	0.005	113	48.6	0.502	34.3	0.342	54.7	0.007
>60 years	282	79.3		74.0		74.8		239	52.0		34.9		41.6	
T classification														
T2	381	–	–	–	–	–	–	150	63.1	< 0.001	48.4	0.000	60.7	<0.001
T3–4	0	–		–		–		202	40.0		23.9		34.9	
N classification														
N0	315	85.3	0.003	80.8	<0.001	82.7	<0.001	69	53.5	<0.001	37.8	0.000	51.2	<0.001
N1	0	–		–		–		224	54.6		38.8		50.7	
N2	0	–		–		–		48	30.8		13.4		22.2	
Nx	66	68.7		56.7		62.6		11	0.0		0.0		0.00	
Preop CA 19-9 level														
≤37 U/mL	254	86.0	0.001	81.1	0.001	83.2	0.026	200	51.9	0.037	35.8	0.046	46.9	0.428
>37 U/mL	51	69.7		61.3		64.3		102	44.5		28.6		43.2	
Postop CA 19-9 level (U/mL)														
≤37	310	84.9	0.000	79.0	0.000	83.7	0.000	252	55.2	<0.001	39.7	0.000	82.4	0.000
>37	17	42.2		47.4		43.6		61	25.6		6.3		14.4	
Histologic differentiation														
WD/MD	329	82.8	0.740	77.2	0.608	78.9	0.964	244	53.4	0.036	38.5	0.004	51.3	0.004
PD	40	84.3		74.3		78.0		90	44.8		25.2		34.1	
Resection margin														
Negative	359	84.1	0.005	79.0	<0.001	81.8	<0.001	311	50.6	0.119	36.2	0.001	48.5	0.001
Positive	10	60.0		40.0		48.0		34	39.1		11.1		18.1	
LVSI														
No	272	87.7	0.000	83.2	<0.001	84.6	<0.001	140	64.6	<0.001	48.5	0.000	66.0	<0.001
Yes	76	63.9		55.8		65.1		196	38.6		23.9		33.3	
PNI														
No	267	87.0	0.000	83.1	<0.001	85.4	<0.001	154	59.6	0.001	44.2	0.000	63.8	0.000
Yes	44	57.2		45.5		49.6		165	40.4		24.2		30.9	
Extent of surgical resection														
Non-radical	79	78.1	0.036	71.3	0.035	64.7	0.009	33	38.7	0.036	34.5	0.335	36.4	0.028
Radical	302	83.9		78.4		83.1		319	51.3		34.6		47.1	
Adjuvant treatment														
No-AT	258	83.9	0.510	79.8	0.076	78.3	0.946	114	37.9	<0.001	28.8	0.006	35.4	<0.001
CTx	82	81.5		72.5		80.3		133	45.0		30.0		45.7	
CRT	41	79.3		69.4		84.3		105	67.8		45.2		56.9	

LRFS, locoregional recurrence-free survival; RFS, recurrence-free survival; OS, overall survival; *n*, number; No-AT, no adjuvant therapy; CTx, chemotherapy; CRT, chemoradiotherapy; Preop, preoperative; Postop, postoperative; CA 19-9, carbohydrate antigen 19-9; WD, well differentiation; MD, moderate differentiation; PD, poor differentiation; LVSI, lymphovascular space invasion; PNI, perineural invasion; Non-radical, cholecystectomy ± lymph node dissection; Radical, radical cholecystectomy and lymph node dissection. *Log-rank test.

**Table 3:** Multivariate analysis of LRFS, RFS, and OS according to tumor stage

Factors	LRFS		RFS		OS	
	HR (95% CI)	*P* value*	HR (95% CI)	*P* value*	HR (95% CI)	*P* value*
Stage II						
Age						
≤ 60 years	1	0.157	1	0.142	1	0.044
> 60 years	2.07 (0.75–5.70)		1.97 (0.79–4.89)		2.78 (1.02–7.53)	
N classification						
N0	1	0.002	1	<0.001	1	0.024
Nx	3.53 (1.57–7.94)		3.70 (1.81–7.58)		2.55 (1.12–5.76)	
Preop CA 19-9 level						
≤37 U/mL	1	0.181	1	0.125	1	0.580
>37 U/mL	1.78 (0.76–4.19)		1.81 (0.84–3.86)		1.30 (0.51–3.29)	
Postop CA 19-9 level						
≤ 37 U/mL	1	0.048	1	0.094	1	0.038
>37 U/mL	3.60 (1.01–12.85)		2.78 (0.84–9.21)		4.27 (1.08–16.91)	
Resection margin						
Negative	1	0.001	1	0.001	1	0.002
Positive	11.11 (2.70–45.65)		10.82 (2.78–42.10)		9.84 (2.25–42.98)	
LVSI						
No	1	0.217	1	0.405	1	0.419
Yes	1.64 (0.74–3.61)		1.35 (0.66–2.78)		1.36 (0.64–2.92)	
PNI						
No	1	0.005	1	<0.001	1	<0.001
Yes	3.25 (1.42–7.40)		3.85 (1.86–7.98)		4.16 (1.91–9.07)	
Extent of surgical resection						
Non-radical	1	0.278	1	0.532	1	0.517
Radical	0.55 (0.18–1.61)		0.74 (0.29–1.87)		0.71 (0.26–1.97)	
Stage III–IV						
Age						
≤ 60 years	1	0.895	0.702	1	0.108
> 60 years	0.97 (0.63–1.48)		1.07 (0.75–1.53)		1.37 (0.93–2.03)	
T classification						
T2	1	0.040	1	0.048	1	0.054
T3–4	1.64 (1.02–2.64)		1.48 (1.00–2.19)		1.51 (0.99–2.32)	
N classification						
N0	1		1		1	
N1	2.02 (1.10–3.68)	0.022	1.77 (1.08–2.91)	0.023	1.68 (0.96–2.94)	0.066
N2	4.30 (2.22–8.29)	<0.001	3.91 (2.24–6.82)	<0.001	4.25 (2.31–7.79)	<0.001
Nx	39.95 (9.33–170.98)	<0.001	21.56 (5.73–81.05)	<0.001	7.85 (1.62–37.84)	0.010
Preop CA 19-9 level						
≤ 37 U/mL	1	0.789	1	0.895	1	0.128
> 37 U/mL	1.06 (0.69–1.62)		0.97 (0.67–1.40)		0.73 (0.49–1.09)	
Postop CA 19-9 level						
≤37 U/mL	1	0.002	1	<0.001	1	<0.001
>37 U/mL	2.18 (1.33–3.56)		2.76 (1.80–4.24)		2.49 (1.59–3.89)	
Histologic differentiation						
WD/MD	1	0.402	1	0.126	1	0.229
PD	1.20 (0.78–1.83)		1.31 (0.92–2.19)		1.26 (0.86–1.85)	
Resection margin						
Negative	1	0.889	1	0.153	1	0.320
Positive	1.05 (0.48–2.31)		1.54 (0.85–2.80)		1.36 (0.74–2.50)	
LVSI						
No	1	0.055	1	0.065	1	0.019
Yes	1.65 (0.98–2.75)		1.46 (0.97–2.19)		1.74 (1.09–2.77)	
PNI						
No	1	0.123	1	0.241	1	0.051
Yes	1.42 (0.90–2.25)		1.25 (0.86–1.81)		1.52 (0.99–2.31)	
Extent of surgical resection						
Non-radical	1	0.010	1	0.169	1	0.259
Radical	0.39 (0.19–0.79)		0.62 (031–1.22)		0.65 (0.30–1.37)	
Adjuvant treatment						
No-AT	1		1		1	
CTx	0.80 (0.50–1.29)	0.366	0.91 (0.60–1.39)	0.687	0.78 (0.51–1.21)	0.280
CRT	0.28 (0.15–0.51)	<0.001	0.52 (0.33–0.82)	0.006	0.42 (0.25–0.70)	0.001

LRFS, locoregional recurrence-free survival; RFS, recurrence-free survival; OS, overall survival; HR, hazard ratio; CI, confidence interval; Preop, preoperative; Postop, postoperative; CA 19-9, carbohydrate antigen 19-9; LVSI, lymphovascular space invasion; PNI, perineural invasion; WD, well differentiation; MD, moderate differentiation; PD, poor differentiation; No-AT, no adjuvant therapy; CTx, chemotherapy; CRT, chemoradiotherapy. *Cox proportional hazards model.

**Figure 3:**
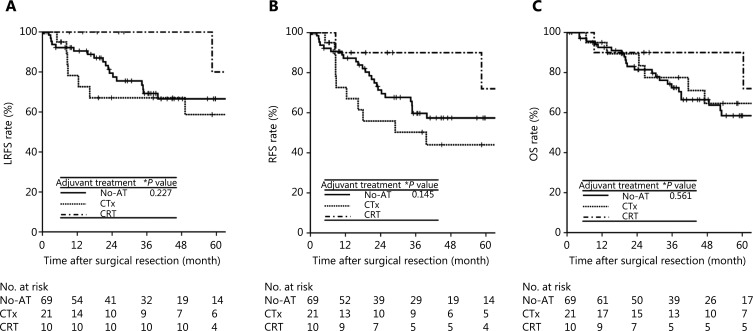
LRFS (A), RFS (B), and OS (C) curves among the adjuvant treatment groups in stage II patients with of perineural invasion and/or Nx classification. LRFS, locoregional recurrence-free survival; RFS, recurrence-free survival; OS, overall survival; LR, locoregional recurrence; DM, distant metastasis; NED, no evidence of disease; No-AT, no adjuvant treatment; CTx, chemotherapy; CRT, chemoradiotherapy. *Log-rank test.

To identify the potential subgroup of patients with stage II disease who are likely to benefit from adjuvant treatments, subgroup analysis was performed using high-risk features, including resection margin (positive *vs.* negative), perineural invasion (presence or absence), and performance of lymphadenectomy (no *vs.* Nx) identified in multivariate analysis. The HRs of positive resection margin for LRFS, RFS, and OS were 11.11, 10.98, and 9.84, respectively, which were higher than those of perineural invasion (3.25, 3.85, and 4.16, respectively) and Nx classification (3.53, 3.70, and 2.55, respectively) (**Table 2**). Of 10 patients with positive resection margins, 3, 4, and 3 patients belonged to the No-AT, CTx, and CRT groups, respectively. There were no significant differences in the event numbers of LRFS [1 (33.3%), 1 (25%), and 2 (66.7%)], RFS [1 (33.3%), 3 (75%), and 2 (66.7%)], and OS [1 (33.3%), 3 (75%), and 2 (66.7%)] in the No-AT, CTx, and CRT groups, respectively (*P* > 0.05). Of 100 patients who had either perineural invasion or Nx classification, 69, 21, and 10 patients belonged to the No-AT, CTx, and CRT groups, respectively. The CRT group had a trend of better 5-year LRFS (80%, 58.7%, and 66.6%), RFS (72%, 44%, and 57.4%), and OS (90%, 64.6%, and 58.4%) than the CTx and No-AT groups, but these differences were not significant due to the small number of patients who underwent CRT (*n* = 10) (**Figure 3**) (*P* > 0.05).

## Discussion

In evaluating the role of adjuvant treatments in resected GBC, a better understanding of the patterns of failures after curative resection could be a prerequisite, but it is still controversial^2,7,8,15–17^. Jarnagin et al.^2^ compared the pattern of failure after curative resection between GBC (*n* = 97) and hilar cholangiocarcinoma (*n* = 80) and reported that recurrences in distant sites were more frequent in GBC than in hilar cholangiocarcinoma (85% *vs.* 41%, *P* < 0.001). Kim et al.^15^ also reported that distant metastasis was a more dominant failure pattern (86%) than locoregional recurrence in resected GBC patients, but they addressed that the information regarding the recurrence pattern was incomplete. In contrast, Park et al.^7^ reported that the most common failure site in resected GBC patients was aortocaval lymph nodes, i.e., 47.4% of all recurrences. Kim et al.^8^ also reported that regional lymph nodes (27.7%) were the most frequent recurrence sites in resected GBC patients. Similarly, in the present study, locoregional recurrence accounted for nearly half of all recurrences in both stages II and III–IV if adjuvant treatments were not administered (**Figure 1**), and these findings suggested that the addition of CRT after curative resection could be a reasonable therapeutic option for resected GBC.

In GBC, disease recurrence after curative resection frequently occurs, ranging from 31.9% to 66.3% in previous studies^2,7,8^ and from 18.2% to 60.5% in ours, but the optimal adjuvant treatment modalities and their efficacy and indications remain unclear. Several studies evaluating the role of adjuvant treatments, including CRT and CTx, have been performed^3,9,12,15,18–22^. Kim et al.^12^ analyzed 151 patients with resected GBC, and adjuvant CRT was associated with significantly better LRFS and OS than CTx and No-AT in T2-3N1-2M0 stage disease, but not in T2-3N0M0. The multi-institutional study by Kim et al.^15^ of 291 resected GBC patients showed that adjuvant CTx/CRT was associated with improved OS (HR, 0.26) compared with No-AT, and the OS benefit by CTx/CRT was significant in patients with high-risk features, such as T3–4 disease (HR, 0.41), positive lymph nodes (HR, 0.45), and microscopic residual disease (HR, 0.21) (*P* < 0.05). Wang et al.^18^ analyzed 1,137 resected GBC patients from the Surveillance, Epidemiology, End Results (SEER) database and proposed a nomogram for predicting OS: CRT and CTx were beneficial in patients with ≥T2 disease, regardless of lymph node involvement, and in patients with T4 or positive lymph node disease, respectively; the OS benefits from CRT for T2-3N0 disease and CTx for T4N0 or positive lymph node disease were small, and those from CRT for T4N0 or positive lymph node disease were large. Similarly, the present study, which included 733 resected GBC patients from a multi-institutional database, also showed that adjuvant CRT significantly improved LRFS, RFS, and OS in patients with stage III–IV disease compared with No-AT and CTx. In addition, although adjuvant CRT/CTx did not provide a significant benefit compared with No-AT in patients with stage II disease, positive resection margin, presence of perineural invasion, and Nx classification were significantly associated with worse LRFS, RFS, and OS, similar to the findings of previous reports^12,15,23^. The 5-year LRFS, RFS, and OS rates in stage II patients with high-risk features, such as positive resection margin, presence of perineural invasion, and Nx classification, were 57.2%–68.7%, 40.0%–56.7%, and 48.0%–62.6%, respectively, which were significantly lower than those (84.1%–87.0%, 79.0%–83.1%, and 81.8%–85.4%, respectively, *P* < 0.05) in stage II patients without these high-risk features and similar to those (67.8%, 45.2%, and 56.9%, respectively) in stage III–IV patients who received CRT (**Table 2**). These findings suggest that stage II resected GBC patients with these high-risk features could be potential candidates for adjuvant treatments, and further studies are warranted.

The role of adjuvant CTx in resected GBC also remains unclear^6,18,24,25^. Subgroup analysis of 112 GBC patients in randomized phase III trials assessing the efficacy of adjuvant CTx using 5-FU and mitomycin C for pancreatobiliary cancer after surgical resection showed that adjuvant CTx improved OS compared with No-AT (5-year OS, 26% *vs.* 14.4%, *P* < 0.05), but most patients in this study had M1 disease (94% and 100% in the CTx and No-AT groups, respectively)^6^. Recently, a phase III randomized controlled trial assessing the clinical outcomes of adjuvant CTx with capecitabine compared with No-AT in 447 resected biliary tract cancer patients, including 79 (17.7%) GBC patients, showed that adjuvant CTx improved OS compared with No-AT in the per-protocol analysis (HR, 0.75; 95% CI, 0.58–0.97; *P* < 0.05), but the OS benefit of adjuvant CTx in the subgroup of GBC was not significant compared with No-AT (HR, 0.84; 95% CI, 0.43–1.63; *P* > 0.05)^25^. A meta-analysis regarding adjuvant treatments for resected bile duct cancer showed that the OS benefit from CTx or CRT was significantly greater than that from radiotherapy alone [odds ratio (OR), 0.39, 0.61, and 0.98, respectively, *P* = 0.02], and the greatest benefit from adjuvant CTx/CRT was in patients with positive lymph nodes (OR, 0.49) and microscopic residual disease (OR, 0.36) (*P* < 0.05)^24^. In contrast, an analysis of SEER data for GBC (*n* = 1,137) showed that the OS benefit from CRT was greater than that from CTx and was prominent in T3–4 disease or positive lymph node disease^18^. Similarly, the present study showed that a statistically significant OS benefit in stage III–IV patients was derived from CRT compared with No-AT but not from CTx. However, in the present study, CRT significantly reduced the rates of locoregional recurrence compared with No-AT and CTx in stage III–IV patients (25.7%, 44.7%, and 41.4%, respectively, *P* = 0.002) but did not significantly reduce the rates of distant metastasis (39%, 41.2%, and 48.9%, respectively, *P* = 0.233). These findings suggest that recurrence at distant sites is still an important failure cause in stage III–IV patients even after CRT, and the addition of CTx to CRT may be meaningful because it effectively reduces distant metastasis.

The present study had inherent limitations due to the use of retrospective data, heterogeneity of treatments in extent of surgical resection, details of radiotherapy and chemotherapeutic regimens, incompleteness of information on performance status and comorbidities of each patient, treatment-related toxicities, and so forth. Probable selection bias, the effect of these confounding factors, and treatment-related toxicities were not thoroughly addressed, but the present study included a relatively large study population (*n* = 733) using a multi-institutional database from KROG to minimize probable bias and the effects of confounding factors, and the heterogeneity of treatments in the present study reflected clinical practice in the real world. Furthermore, the present study included 112 (15.3%) patients who underwent cholecystectomy rather than radical cholecystectomy and 77 (10.5%) patients who did not undergo lymph node dissection, and these may lead to incorrect staging. Thus, in the present study, the extent of surgical resection and N classification were evaluated, and the prognostic significance for LRFS, RFS, and OS and Nx classification was consistently independent significant prognostic factor associated with LRFS, RFS, and OS in patients with stages II and III–IV (**Tables 2 and 3**). These findings suggest that the completeness of surgical resection is an important factor related with prognosis of GBC patients in the real world. In addition, although several studies evaluating the role of adjuvant treatments for resected GBC patients using SEER and/or multi-institutional databases have been performed^18,20,21^, the present study compared the patient characteristics, patterns of failures, LRFS, RFS, and OS among adjuvant treatment groups according to stage and attempted to identify the subgroup most likely to benefit from adjuvant treatments, including CRT and/or CTx.

## Conclusions

The present study showed that CRT significantly improved the LRFS, RFS, and OS of resected GBC patients with stage III–IV disease. In addition, the LRFS, RFS, and OS rates of stage II patients with high-risk features, such as positive resection margin, presence of perineural invasion, and Nx classification, were similar to those of stage III–IV patients receiving adjuvant CRT, and recurrence at distant sites was one of the main patterns of failures in stage III–IV, even after adjuvant CRT. Although the present study did not show the OS benefit of adjuvant CRT in stage II patients and the addition of CTx to CRT in stage III–IV patients, these findings suggest that further studies on CRT for stage II patients with high-risk features and the addition of CTx to CRT for stage III–IV patients are warranted.
